# G6PD is a prognostic biomarker correlated with immune infiltrates in lung adenocarcinoma and pulmonary arterial hypertension

**DOI:** 10.18632/aging.205381

**Published:** 2024-01-08

**Authors:** Rongzhen Ding, Shuliu Sang, Jian Yi, Haiping Xie, Feiying Wang, Aiguo Dai

**Affiliations:** 1 Department of Respiratory Diseases, Medical School, Hunan University of Chinese Medicine, Changsha, China; 2 Hunan Provincial Key Laboratory of Vascular Biology and Translational Medicine, Changsha, China; 3 Department of Respiratory Medicine, First Affiliated Hospital, Hunan University of Chinese Medicine, Changsha, China; 4 Department of Urinary Surgery, First Affiliated Hospital, Hunan University of Chinese Medicine, Changsha, China; 5 Shanghai University of Traditional Chinese Medicine, Shanghai, China; 6 Hunan Academy of Chinese Medicine, Changsha, China

**Keywords:** G6PD, immune cells, lung adenocarcinoma, pulmonary arterial hypertension, WGCNA

## Abstract

Background: Lung adenocarcinoma (LUAD) with Pulmonary arterial hypertension (PAH) shows a poor prognosis. Detecting related genes is imperative for prognosis prediction.

Methods: The gene expression profiles of LUAD and PAH were acquired from The Cancer Genome Atlas (TCGA) and the Gene Expression Omnibus (GEO) database, respectively. The co-expression modules associated with LUAD and PAH were evaluated using the Weighted Gene Co-Expression Network Analysis (WGCNA). The relationship between key gene expression with immune-cell infiltration and the tumor immune microenvironment (TIME) was evaluated. We confirmed the mRNA and protein levels *in vivo* and *vitro*. G6PD knockdown was used to conduct the colony formation assay, transwell invasion assay, and scratch wound assay of A549 cells. EDU staining and CCK8 assay were performed on G6PD knockdown HPASMCs. We identified therapeutic drug molecules and performed molecular docking between the key gene and small drug molecules.

Results: Three major modules and 52 overlapped genes were recognized in LUAD and PAH. We identified the key gene G6PD, which was significantly upregulated in LUAD and PAH. In addition, we discovered a significant difference in infiltration for most immune cells between high- and low-G6PD expression groups. The mRNA and protein expressions of G6PD were significantly upregulated in LUAD and PAH. G6PD knockdown decreased proliferation, cloning, and migration of A549 cells and cell proliferation in HPASMCs. We screened five potential drug molecules against G6PD and targeted glutaraldehyde by molecular docking.

Conclusions: This study reveals that G6PD is an immune-related biomarker and a possible therapeutic target for LUAD and PAH patients.

## INTRODUCTION

Lung adenocarcinoma (LUAD), accounts for approximately 40% of all lung cancer cases [1], has become the most frequent clinical histological subtype of lung cancer [2]. Since the early prevention and variety of lung cancer therapy, the therapeutic effect for lung cancer patients has significantly improved [3]. A few studies have reported that lung cancer patients tend to develop symptoms of dyspnea, parenchymal disease, or left heart disease with pulmonary hypertension (PH), which affects their quality of life [4, 5].

PH is frequently related to chronic lung diseases such as chronic obstructive pulmonary disease, interstitial lung disease, and lung cancer [6–8]. Patients with lung cancer and different animal models constructed by lung cancer-associated genes (LCC1, cRaf-BxB, KRasLA2) were reported to have pulmonary vascular remodeling and PH [9]. Another study reported that 43.7% of lung cancer patients had a mean pulmonary artery (PA) diameter less than 28 mm, implying that lung cancer may cause PH [10]. Pulmonary arterial hypertension (PAH), a primary arteriolar vasculopathy in PH, is a progressive obliterative vasculopathy and usually causes right ventricular failure and death [11]. Therefore, discovering the correlation and shared pathogenesis between LUAD and PAH is urgently needed.

Even though few research concentrated on the exact cellular and molecular evidence between LUAD and PAH, previous research revealed that they have common risk factors and pathogenesis [12, 13]. Smoking was reported to be the common factor in the two diseases [14, 15]. The pathophysiological mechanisms, including glycolysis and mitochondrial metabolism, were identified for the interplay between LUAD and PAH [16, 17]. Glycolysis was considered to promote LUAD proliferation and vascular remodeling in PAH [18, 19]. 3-Bromopyruvate, a glycolytic inhibitor, improved mitochondrial metabolism, thereby repressing pulmonary artery smooth muscle cells (PASMCs) proliferation and effectively relieving PAH [20]. Meanwhile, LUAD tumor growth is inhibited by targeting mitochondrial trifunctional proteins [21]. Moreover, the study has specified that Metastasis-Related Lung Adenocarcinoma Transcript 1 could regulate the expression of Toll-like receptor 4 (TLR4) with increased cell proliferation and migration in PASMCs [22]. These findings strongly suggest that LUAD and PAH are interrelated. However, these studies are little about the common features of mechanisms at the molecular level.

Contemporary, the rapid advances in bioinformatics approaches allow us to delve deeper into the molecular-level investigation of disease-disease interactions and co-pathogenesis [23–26]. To discover the biological mechanisms of LUAD and PAH, we used the weighted gene co-expression network analysis (WGCNA) to discover the co-expression cluster and connected shared genes in these two diseases and explored potential drug molecules using molecular docking. This study lays the foundation for future clinical challenges between LUAD and PAH with systems biology strategy.

## MATERIALS AND METHODS

### Data download and process

The keyword “pulmonary arterial hypertension” was used to search PAH gene expression profiles in the Gene Expression Omnibus (GEO) datasets (http://www.ncbi.nlm.nih.gov/geo/) [27], and the mRNA expression data for LUAD was acquired from The Cancer Genome Atlas (TCGA) data portal (https://www.cancer.gov/tcga). These datasets from the GEO public database were screened based on the four inclusion criteria: (I) All datasets should include a cases-control group. (II) All samples should be available from human lung tissues. (III) The range of samples per group n>10 ought to be acquired to determine the accuracy of the results of the WGCNA. (IV) The original data in these databases could be processed and analyzed. The GEO datasets, GSE15197, GSE113439, and GSE32867 were selected. From the TCGA and GEO databases, 673 LUAD samples and 432 normal samples were retrieved. We gathered 33 PAH samples and 24 normal samples from the GEO database. Gene expression profiling data were log2-transformed for further investigation.

### Construction of co-expression network

WGCNA, a systematic biology algorithm, aims to construct gene co-expression networks and describes correlations among genes across multiple. For the present study, we ran the R package “WGCNA” to estimate LUAD and PAH modules of the correlated gene. Before analysis, the outliers were excluded via the cutreeStatic function. Then, the adjacency matrix was generated by soft threshold b (6 for LUAD, 19 for PAH) to fit the network structure best, and the gene-gene correlation matrix was constructed by computing Pearson correlations between gene expression levels to the connectivity between the nodes. Next, using a hierarchical clustering dendrogram of the matrix, a topological overlap matrix (TOM) was built to separate different modules following similar gene expressions. Finally, we identified the module eigengene (ME) expression profiles by compiling the expression profiles for each module to look for a link between ME and clinical status. The candidate modules were screened with a significant correlation coefficient with clinical traits.

### Enrichment analysis of the shared genes in LUAD and PAH

After the co-expression network was constructed, we chose the positive correlation coefficients modules of LUAD and PAH to overlap the shared genes and visualized them by using the Jvenn. Then, we analyzed these shared gene functional annotations using GO and KEGG enrichment analysis. KEGG was utilized to associate genomic information with function for systematic analysis [28]. GO was utilized to analyze gene function, and the enrichment terms with a *P*-value < 0.05 were considered significant [29].

### Overlapped gene targets obtained from disease database and WGCNA

GeneCards is a disease database that includes all annotated information on human genes [30]. We used the GeneCards disease database to screen the gene targets of LUAD and PAH, respectively. Then, the disease genes from GeneCards and the co-expressed genes analyzed by WGCNA were intersected.

### Protein-protein interactions network construction

To establish a protein interactions network, we employed the Search Tool for Retrieval of Interacting Genes (STRING) web tool [31]. Overlapped genes were imported into STRING with the confidence score set to 0.4. After that, the CytoHubba Cytoscape plugin was applied to select the hub genes from the protein-protein interaction (PPI) network. Here, the 12 algorithms of CytoHubba (Betweenness, Stress, Radiality, Eccentricity, Degree, DMNC, EPC, MCC, Closeness, MNC, Clustering Coefficient, and BottleNeck) were chosen. Using the GeneMANIA online tool (http://www.genemania.org/) [32], we constructed a co-expression network involving the hub genes.

### Cell culture

LUAD cell lines (A549, H1975, H460, PC9) and BEAS 2B cells were acquired from the Cell Bank of the Chinese Academy of Sciences and Human Pulmonary Arterial Smooth Muscle Cells (HPASMCs) were obtained from ATCC. A549, H1975, H460, PC9, and BEAS 2B cells were grown in RPMI-1640 with 10% fetal bovine serum supplement in a 37° C incubator with 5% CO_2_. HPASMCs were grown in DMEM medium with 10% fetal bovine serum. Then, the cells were planted in a 37° C humidified incubator with 3% O_2_ for 24h to construct a hypoxia cell model [33]. The CCK-8 test was used to confirm the hypoxia HPASMCs model.

### Hub genes validation in LUAD and PAH

To confirm the key gene in LUAD and PAH, we performed the expression of hub genes on discovery datasets (TCGA and GSE15197) and validation datasets (GSE113439 and GSE32867) via the Wilcoxon rank-sum test and ggplot2 with cutoff *P*-value <0.05, respectively. We determined the intersection of hub genes in the discovery and validation cohort. Subsequently, we acquired immunohistochemistry (IHC) images of LUAD and normal tissues via the Human Protein Atlas (HPA) portal [34]. Moreover, we also performed the prognostic analysis of key genes in LUAD patients. GSEA was carried out to calculate the enrichment scores for comparing the high- and low- groups in accordance with key gene median expression. The false discovery rate (FDR) <0.05 was significant. Moreover, we performed the single sample gene set enrichment analysis (ssGSEA) to determine the associated biological pathways.

### Key gene and immune profile analysis

To investigate the associations between the expression of the key gene and immune infiltration, we carried out correlation analyses. The CIBERSORT algorithm in R software was used to calculate 22 immune cell types in patients with distinct immunological patterns, and we used box plots to show possible associations between distinct classes of immune cell infiltration and hub gene. Moreover, the abundance of 28 immune cell types and immune-cell pathway enrichment in individuals with various immunological patterns were determined using the ssGSEA method and the R package "GSVA". Patients were separated into two groups based on the median values of key gene expression levels to determine the degree of immune cell infiltration. The Wilcoxon rank-sum test was applied to evaluate differences in immune cell proportions, and the *P* < 0.05 was deemed statistically significant. Additionally, we compared the expression of immune checkpoints in two groups. Finally, we evaluated the immune and stromal components in the tumor immune microenvironment (TIME) by the R package “estimate”.

### scRNA sequencing data and hallmarks of cancer analysis

Single-cell RNA sequencing (scRNA-seq) data of LUAD patients were acquired from the GEO database numbered GSE117570 and analyzed by the “Seurat” package. After that, the immune cell clusters were identified through the “FindNeighbors” and “FindClusters” functions. We assessed the expression of the key gene in the same type of immune cells between high- and low- groups using the “FindAllMarkers” function. The raw RNA-seq data of the 50 hallmarks of cancer gene sets were scanned in GSEA. The expression of the key gene in the 50 hallmarks of cancer gene sets between two groups was quantified by GSVA and visualized in a heat map.

### qRT-PCR validation of the key gene

We isolated the total RNA from cells via TRIzol reagent (Invitrogen, USA). For cDNA synthesis, a cDNA Synthesis kit (Invitrogen, Thermo Fisher Scientific Inc., USA) was performed to reverse the transcription reaction into cDNA. The relative mRNA levels were assessed by the 2^-ΔΔCq^ calculation method and normalized by GAPDH mRNA expression. Primers were as follows: G6PD forward primers: AACATCGCCTGCGTTATCCTC, reverse primers: ACGTCCCGGATGATCCCAA. GAPDH forward primers: CAATGACCCCTTCATTGACC, reverse primers: GACAAGCTTCCCGTTCTCAG.

### The xenograft tumor model and hypoxia-induced PAH model establishment

Six-week-old female BALB/c nude mice (Hunan SJA Laboratory Animal Co., Ltd., China) were chosen to establish the xenograft tumor model. A549 cells (5×10^5^) were injected subcutaneously under the right flank. Male Sprague Dawley (SD) rats and mice (the Company of Experimental Animals of Hunan Slilaike Jingda, China) were used to establish a hypoxia-induced PAH model, which was exposed to normobaric hypoxia (10% oxygen environment) for 3 weeks as previously described [35].

### Immunofluorescence staining

At 21 days, all mice were put to death by cervical dislocation, and the tumors and lung organs were taken out from BALB/c nude mice. Hypoxia-induced PAH and normal lung tissue were taken out from the SD rats and mice. All the organs’ sections were treated with G6PD antibodies (1:1000, Abcam, USA) overnight at 4° C. Then, the sections were exposed to a FITC-conjugated secondary antibody (1:1000) for 2 h. Finally, samples were visualized using a fluorescent inverted microscope (IX73-A22FL/PH; Olympus Corporation, Japan; light source: UHP).

### Transfection

G6PD knockdown was achieved by G6PD coding siRNA transfection via Lipofectamine® 2000 reagent (Invitrogen, USA).

### EDU assay

Approximately 3,000 transduced hypoxia-treated HPASMCs and normal HPASMCs were cultivated in 96-well plates. The cells were labeled with EDU for 2h, and then the labeling efficiency in EDU was observed using fluorescence microscopy.

### CCK8 assay

CCK8 assay was applied to measure the proliferative abilities of transduced hypoxia-treated HPASMCs and normal HPASMCs. 3,000 cells/well were plated in 96-well plates and incubated for 48h. Afterward, 10 μL of CCK-8 reagent was applied to each well and the Optical density (OD) values were measured at 450nm within 1-4 hours.

### Colony formation

Colony formation was applied to measure the proliferative abilities of transduced A549 cells and A549 cells. Briefly, 1,000 cells/well were seeded into 6-well plates and incubated for 14 days. When visible colonies appeared, Cells were fixed and stained using 4% paraformaldehyde and crystal violet (Sangon, China).

### Transwell invasion assay

The upper transwell chambers with Matrigel (Corning, USA) were applied to evaluate the invasive property. Approximately 2×10^4^ transduced A549 cells and A549 cells were cultivated in the upper chambers using RPMI without FBS, and the lower chamber was refilled with RPMI containing 10% FBS. The invaded cells were fixed with 4% paraformaldehyde and crystal violet before the invasions were measured.

### Scratch wound assay

Cell migration was applied via scratch wound assay. Transduced A549 cells and A549 cells were seeded into 12-well plates with 80% confluence, and scratch wounds were scraped by a 20 μl tip and photographed after 24 h.

### Potential drug molecules screening and molecular docking simulation

DSigDB database was utilized to link gene proteins to drugs/compounds for drug development research [36]. For exploring the direct link between potential drug molecules for LUAD and PAH with the key gene, the key gene was imported into the Enrichr database (https://amp.pharm.mssm.edu/Enrichr/) [37] to access the DSigDB database and the drug molecules associated with the key gene were downloaded. We ranked these drug molecules by the adjusted *P*-value (*P*<0.05) and screened out the top five molecules.

We further utilized the PubChem database [38] to perform the 3D structures of these molecules and minimized their energy via Chem 3D to simulate how the five drug molecules bind to the key gene. Then, the highest-resolution protein crystal structure of the key gene was acquired from the Protein Data Bank (PDB) databases (https://www.rcsb.org) [39]. After dehydration and hydrogenation through Discovery Studio 2016, we performed molecular docking using the CDOCKER method and calculated binding energy to explore potential interactions between five drug molecules and the key gene.

### Statistical analysis

All statistical analysis and visualization were conducted via GraphPad Prism 8.0 software and R.4.1.1. We determined the group disparities using the Student's t-test. *P* < 0.05 was considered significant.

### Data availability statement

Publicly available datasets are available from the GEO datasets (http://www.ncbi.nlm.nih.gov/geo/) and TCGA data portal (https://www.cancer.gov/tcga).

## RESULTS

### Data information

**Figure 1:**
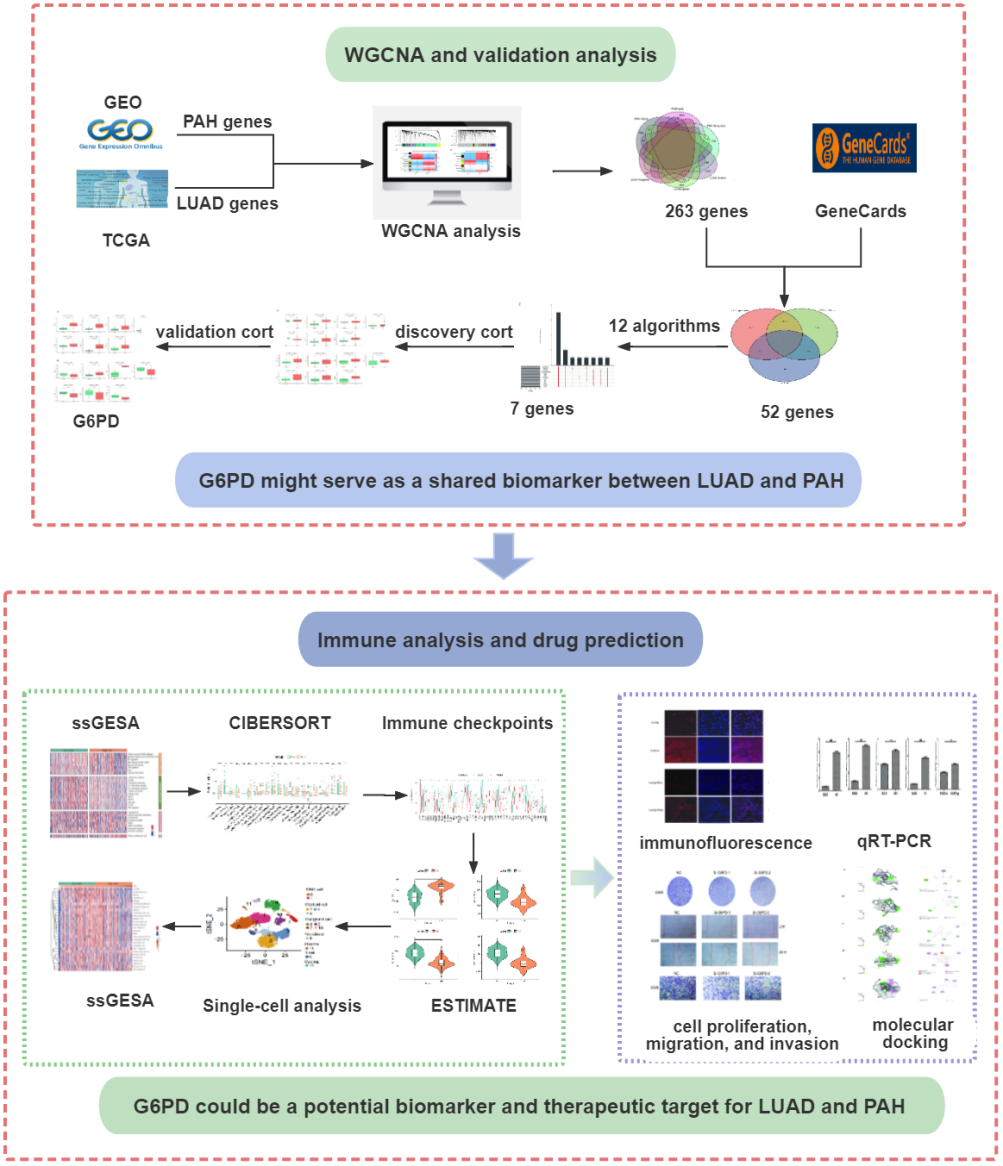
**The flowchart of the present study design.** LUAD, lung adenocarcinoma; PAH, pulmonary arterial hypertension.

A detailed flowchart of this study is presented in Figure 1. We selected GEO datasets numbered GSE15197, GSE113439, and GSE32867. The details of the selected datasets were displayed in Table 1. For the WGCNA analysis, we further chose GSE15197 as a discovery cohort and GSE113439 and GSE32867 as validation cohorts. Meanwhile, the TCGA database was applied to WGCNA analysis as a discovery cohort for LUAD.

**Table 1:** Data information.

**ID**	**Dataset information**	**Samples**	**Disease**	**Group**
1	GSE15197	18 patients and 13 controls	PAH	Discovery
2	TCGA	528 patients and 288 controls	LUAD	Discovery
3	GSE113439	15 patients and 11 controls	PAH	Validation
4	GSE32867	145 patients and 144 controls	LUAD	Validation

### Co-expression modules in LUAD and PAH

**Figure 2:**
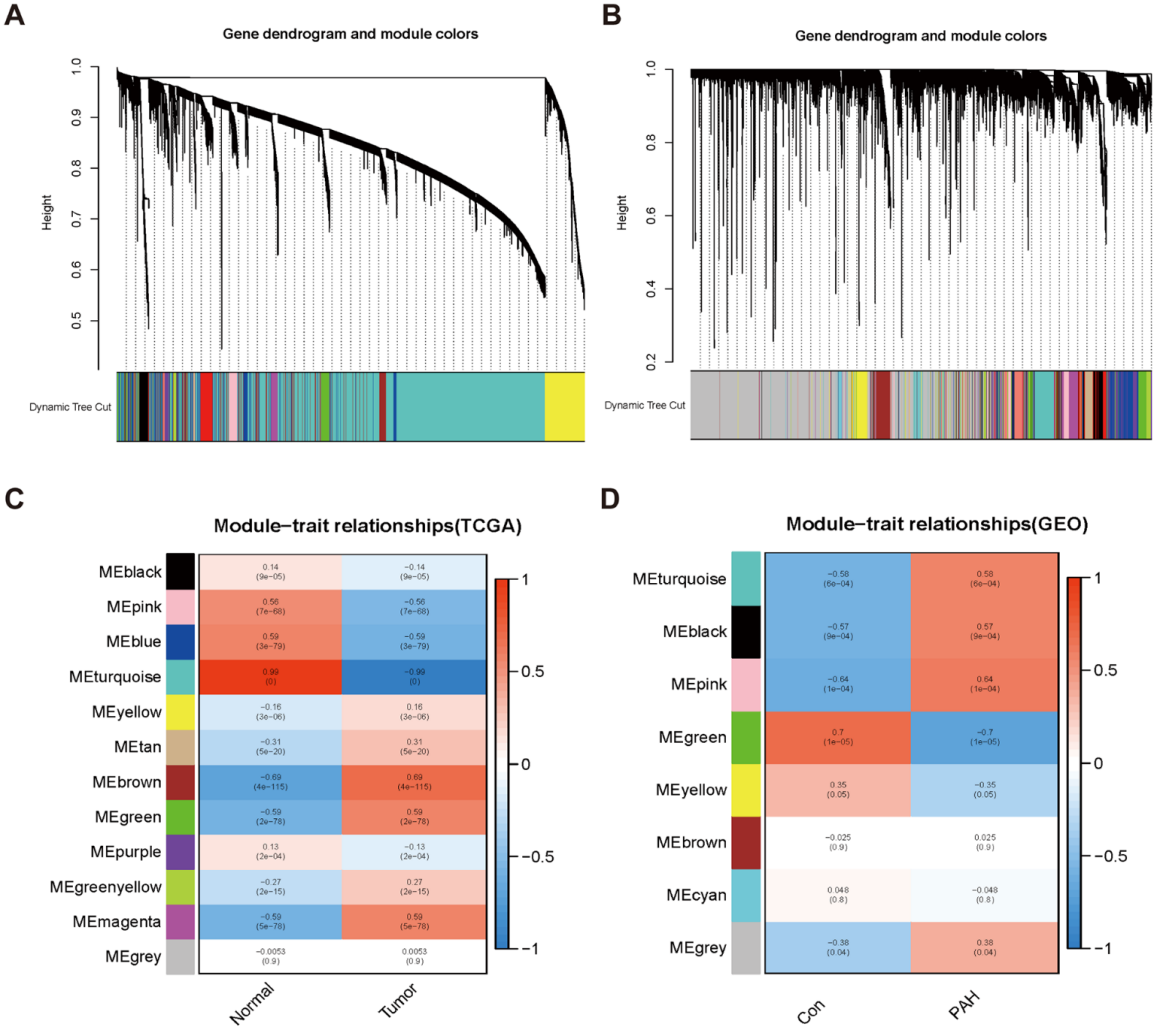
**Consensus module analysis of LUAD and PAH using WGCNA.** (**A**, **B**) Cluster dendrogram of LUAD (**A**) and PAH (**B**). (**C**, **D**) Heap of module–trait relationships in LUAD (**C**) and PAH (**D**).

Twelve modules were generated in TCGA by WGCNA, and different colors represented different modules. Then, we mapped the heat map which could assess the association between modules and the disease based on the Spearman correlation coefficient (Figure 2A, 2C). We selected the modules “magenta,” “green,” and “brown” as LUAD-related modules, which were highly positively correlated with LUAD (magenta module: r = 0.59, p = 5e−78, genes = 245; green module: r = 0.59, p = 2e−78, genes = 516; brown module: r = 0.69, p = 4e−115, genes = 1899). Similarly, eight modules were identified in GSE113439, and the module “black” (r = 0.57, p = 9e−04, genes = 853), “pink” (r = 0.64, p = 1e−04, genes = 784) and “turquoise” (r = 0.58, p = 6e−04, genes = 767) were highly positively related to PAH (Figure 2B, 2D).

### Enrichment analyses of shared genes and identification of overlapped genes in LUAD and PAH

**Figure 3:**
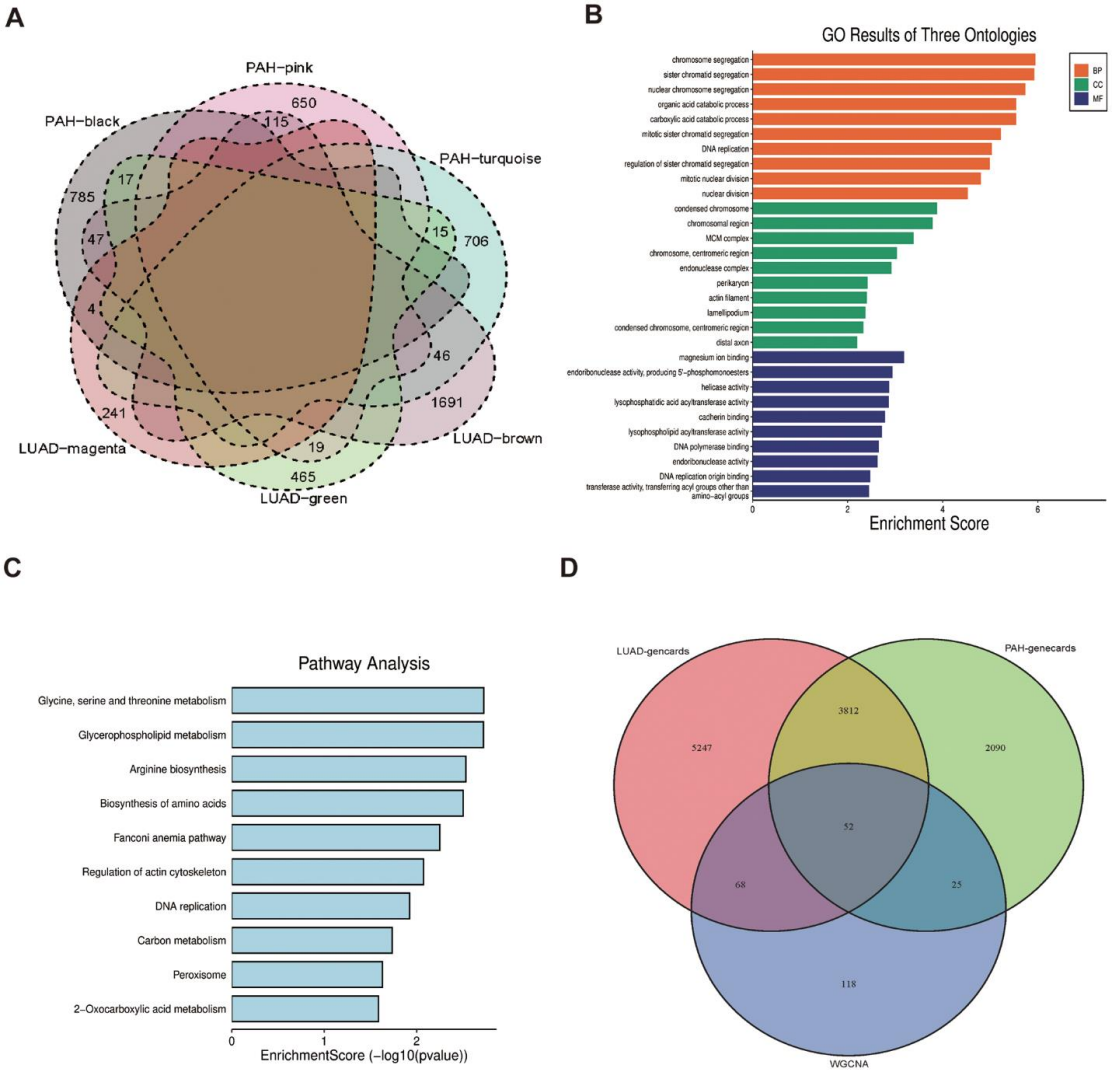
**Functional enrichment based on 263 shared genes.** (**A**) Venn diagram of the shared genes between the magenta, green and brown modules of LUAD and the black, pink, and turquoise modules of PAH. (**B**) GO analysis for 263 shared genes indicating the significant terms. (**C**) KEGG analysis for 263 shared genes. (**D**) Venn diagram of the overlapped genes between GeneCards databases and WGCNA.

The 263 genes in the intersection were positive-correlated modules of LUAD and PAH and were considered to be connected with the pathogenesis of LUAD and PAH (Figure 3A). To investigate biological data of the shared genes in LUAD and PAH, the GO functional and KEGG analyses were carried out. The results indicated that GO categories mainly enriched in chromosome segregation, sister chromatid segregation, nuclear chromosome segregation, etc. (Figure 3B). Additionally, we found that the KEGG categories are related to metabolism (Figure 3C). To further determine the biological functions and find hub genes related to LUAD and PAH, we used GeneCards databases and selected 9179 genes associated with LUAD and 5979 genes associated with PAH. Moreover, 52 overlapped genes between LUAD and PAH were mapped after taking the intersection of GeneCards and WGCNA, which hinted that LUAD and PAH shared common genes (Figure 3D). The list of 52 overlapped genes of LUAD and PAH was shown in the Supplementary Table 1.

### Enrichment analyses of overlapped genes

**Figure 4:**
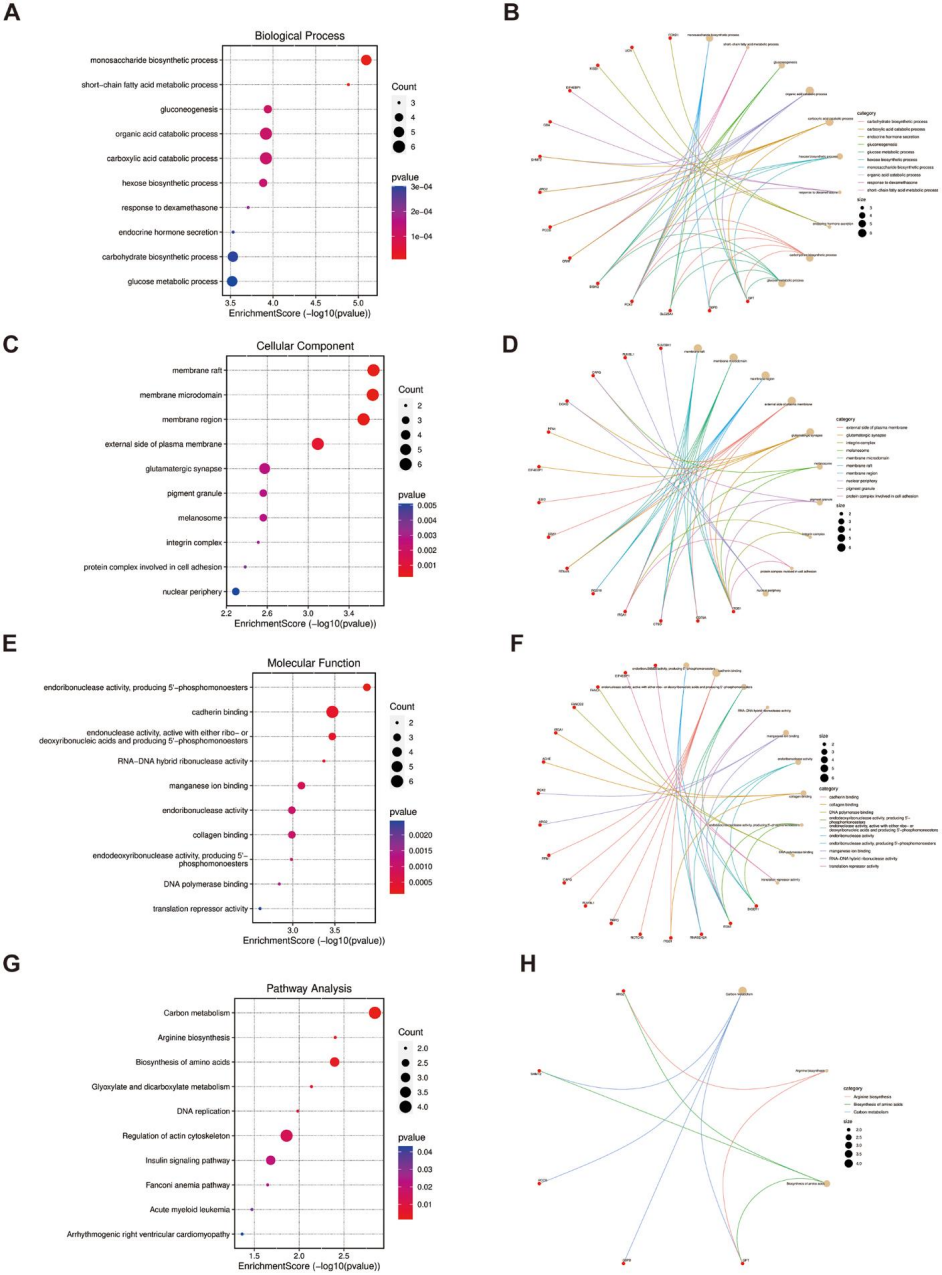
**Functional enrichment based on 52 overlapped genes.** (**A**, **B**) BP analysis and specific genes related to the BP terms. (**C**, **D**) CC analysis and specific genes related to the CC terms. (**E**, **F**) MF analysis and specific genes related to the MF terms. (**G**, **H**) KEGG analysis and specific genes related to these pathways. BP, biological process; CC, cellular component; MF, molecular function.

As for the 52 overlapped genes, we found they were associated with several metabolisms, such as monosaccharide biosynthetic process, gluconeogenesis, and short-chain fatty acid metabolic process by GO terms (Figure 4A, 4C, 4E), while enriched KEGG pathway enrichment entries contained carbon metabolism, arginine biosynthesis and biosynthesis of amino acids, etc. (Figure 4G). Moreover, the cnetplots were used for the specific GO terms and KEGG categories (Figure 4B, 4D, 4F, 4H). We noted that GO functions were significantly enriched in metabolism lay compared to the previous enrichment analyses of shared genes in LUAD and PAH.

### PPI network construction and the hub gene selection

**Figure 5:**
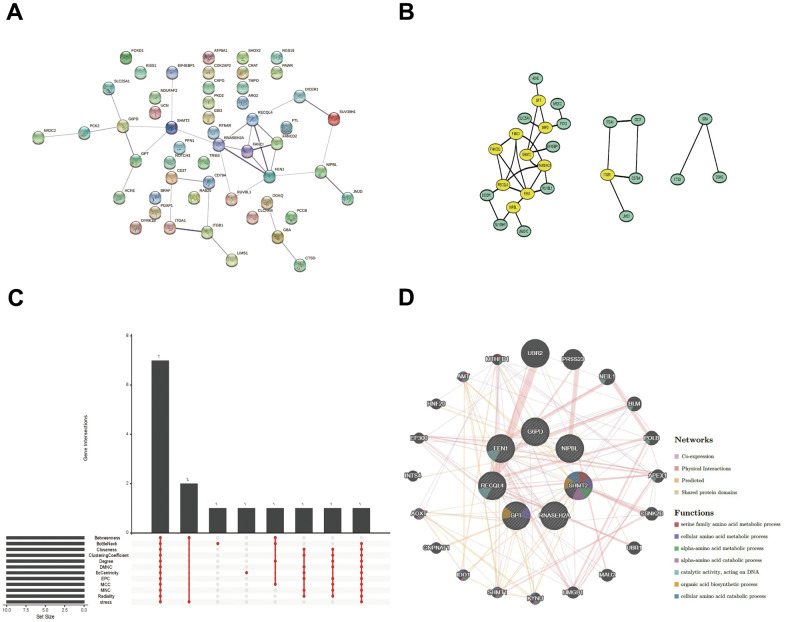
**PPI network, Venn diagram, and co-expression network analysis.** (**A**, **B**) The PPI network of the 52 overlapped genes through the STRING database. (**C**) The Venn diagram indicated that 7 hub genes were screened out by 12 algorithms. (**D**) Co-expression network of 7 hub genes was shown by GeneMANIA.

For a further selection of the hub gene in LUAD and PAH, A PPI network was constructed utilizing Cytoscape software with the confidence interaction score exceeding 0.4 (Figure 5A, 5B). To understand the interactions among the key gene, CytoHubba’s 12 algorithms were applied to calculate 7 hub genes including G6PD, F1N1, RECQL4, GPT, RNASEH2A, SHMT2, and NIPBL (Figure 5C). The co-expression network and associated functions of the seven hub genes were analyzed by the GeneMANIA database. The complex PPI network of seven hub genes included co-expression, physical interaction, prediction, and shared protein structural domains with percentages of 41.08%, 39.10%, 13.30%, and 6.52%, respectively (Figure 5D).

### Immune cell infiltration evaluation of key gene

**Figure 6:**
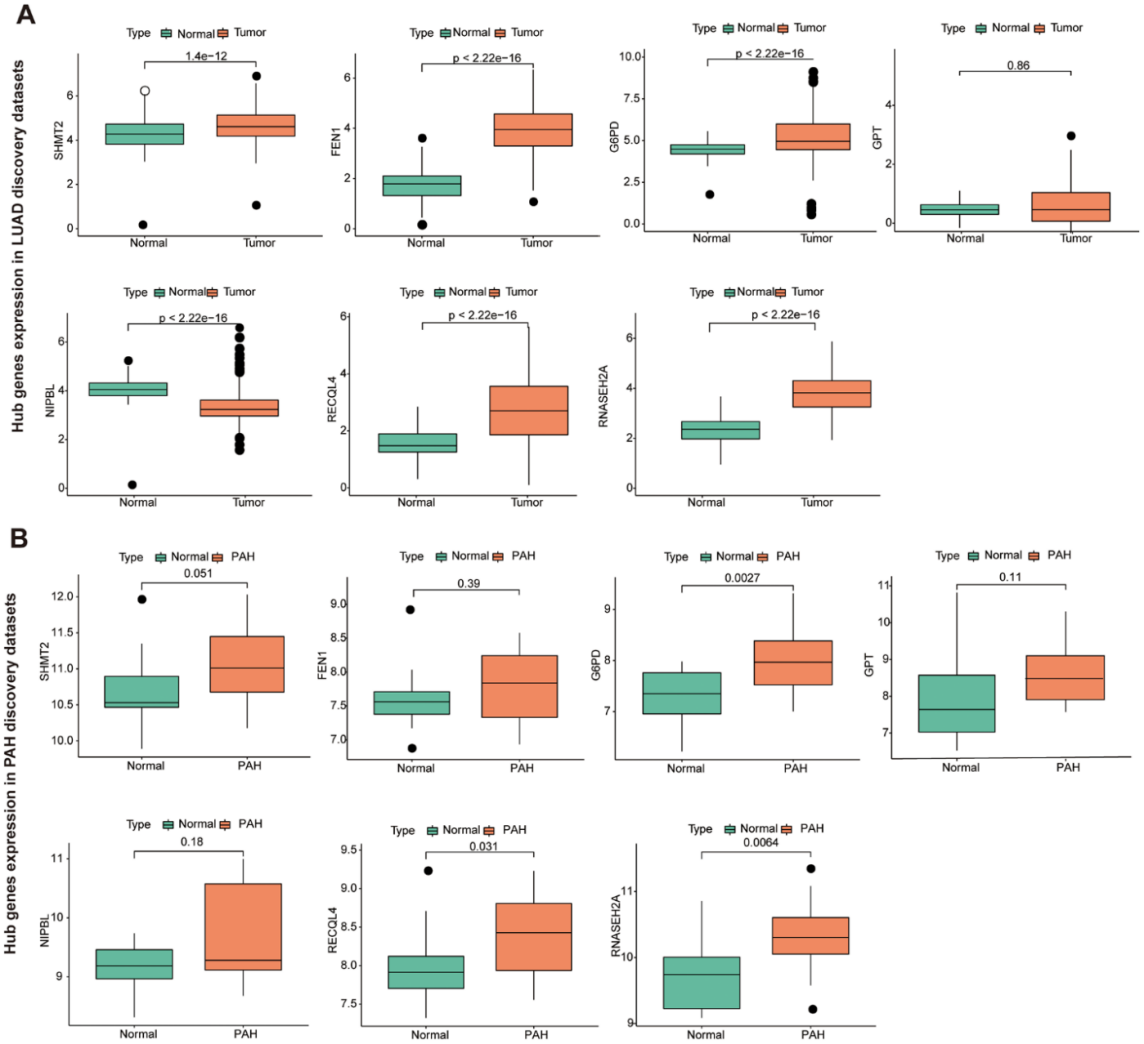
**The hub genes in LUAD and PAH discovery cohorts.** (**A**) The hub genes expression in LUAD discovery cohorts. (**B**) The hub genes expression in PAH discovery cohorts.

**Figure 7:**
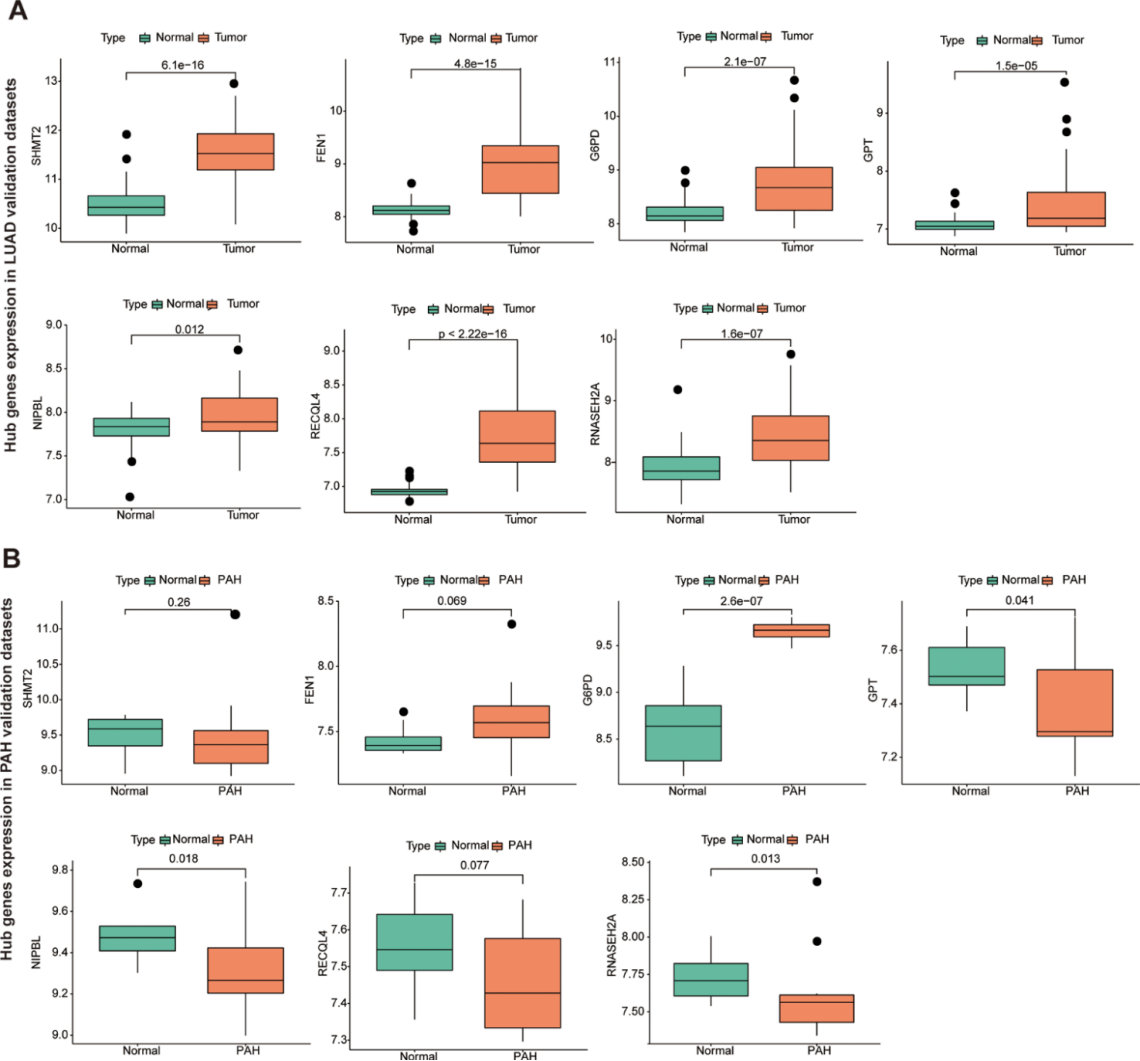
**The hub genes in LUAD and PAH validation cohorts.** (**A**) The hub genes expression in LUAD validation cohorts. (**B**) The hub genes expression in PAH validation cohorts.

**Figure 8:**
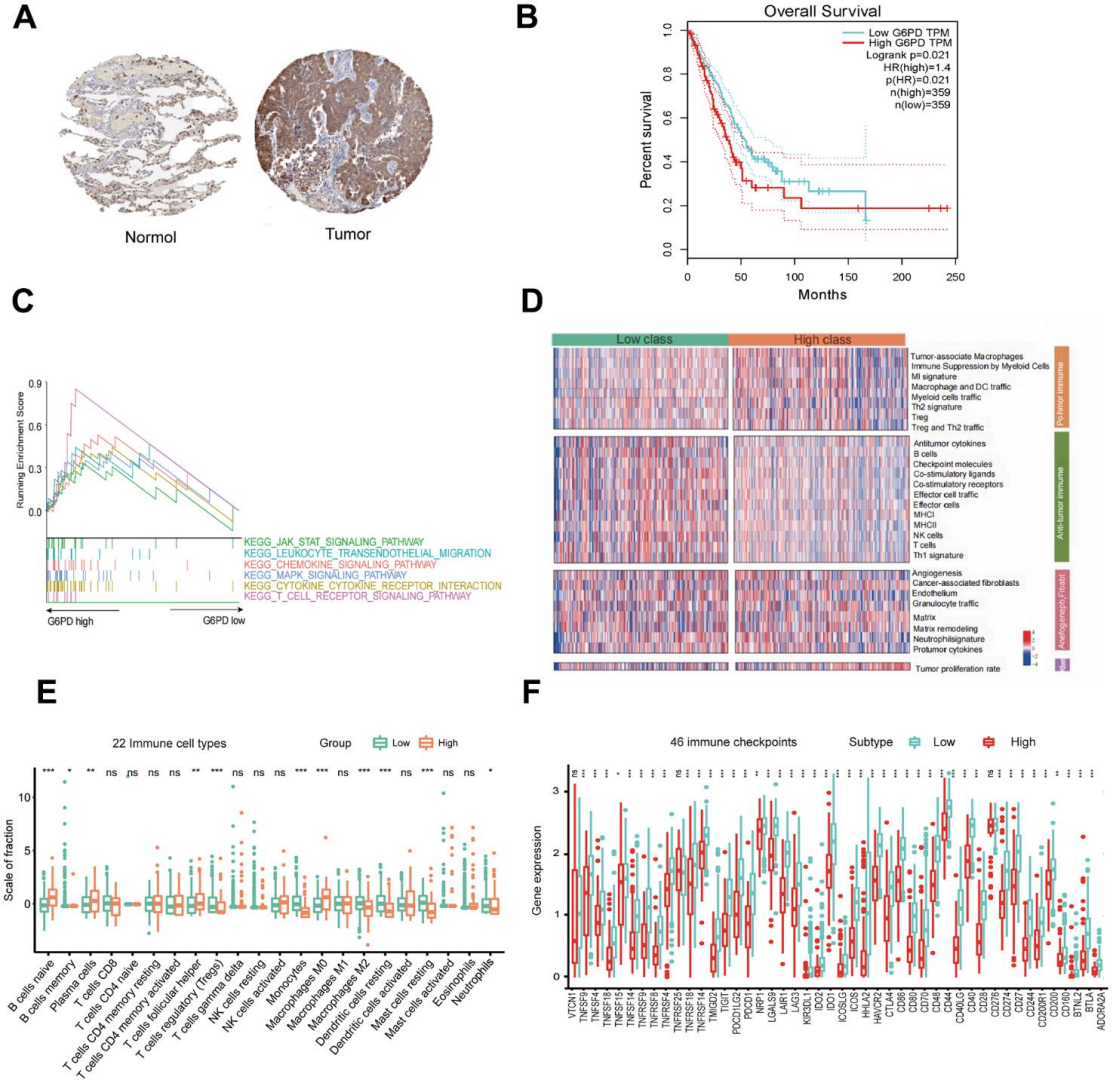
**G6PD expression, prognostic value, and enriched pathways in LUAD tissues.** (**A**) IHC staining of G6PD in normal and LUAD tissues from the HPA database. (**B**) The prognostic analysis of G6PD in LUAD patients. (**C**) GSEA of the enriched pathways in LUAD patients with high G6PD expression (**D**) The enriched immune-related pathways in LUAD patients by ssGSEA. (**E**) The level of immune cell infiltration of 22 subpopulations of immune cells in the G6PD high and low groups based on the CIBERSORT algorithm. (**F**) The levels of immune checkpoint molecules in the G6PD high and low groups.

To further identify the key gene, we analyzed the 7 hub genes between cases and controls on discovery cohorts. As shown in Figure 6, three genes were selected, including G6PD, RECQL4, and RNASEH2A. Meantime, G6PD, GPT, NIPBL, and RNASEH2A were found in validation cohorts (Figure 7). Finally, we found that the GP6D was upregulated in both LUAD and PAH based on discovery and validation cohorts with *P* <0.05. In addition, GP6D had a higher expression in LUAD samples than normal samples through the HPA database (Figure 8A), and the high G6PD expression was related to an unfavorable overall survival (OS) (Figure 8B) by prognostic analysis. GSEA results showed that G6PD over-expression was related to immune-related pathways, such as the T cell receptor signaling pathway and chemokine signaling pathway (Figure 8C). Meanwhile, ssGSEA results indicated that G6PD was enriched in the immune-related pathway (Figure 8D). We found that G6PD participated in the immune regulation of human cancers via TISIDB database [40] (Supplementary Figure 1). Then, we investigated the degree of immune cell infiltration in 22 immune cell subpopulations with high and low G6PD gene expression using the CIBERSORT algorithm. CIBERSORT analysis revealed that the high expression group had significantly lower levels of memory B cells, regulatory T cells, monocytes macrophages M2, resting mast cells, and resting dendritic cells than those in the low group. However, the levels of naive B cells, plasma cells, follicular helper T cells, macrophages M0, and neutrophils were significantly higher than those in the low group (Figure 8E). Besides, we compared the expression levels of immune checkpoints between the G6PD high and low groups and further found that patients in the high group had increased the expression of TIGIT, CTLA4, CD40, CD274, and PDCD1, etc. (*P* < 0.001) (Figure 8F).

### Evaluation of TIME and single-cell analysis

**Figure 9:**
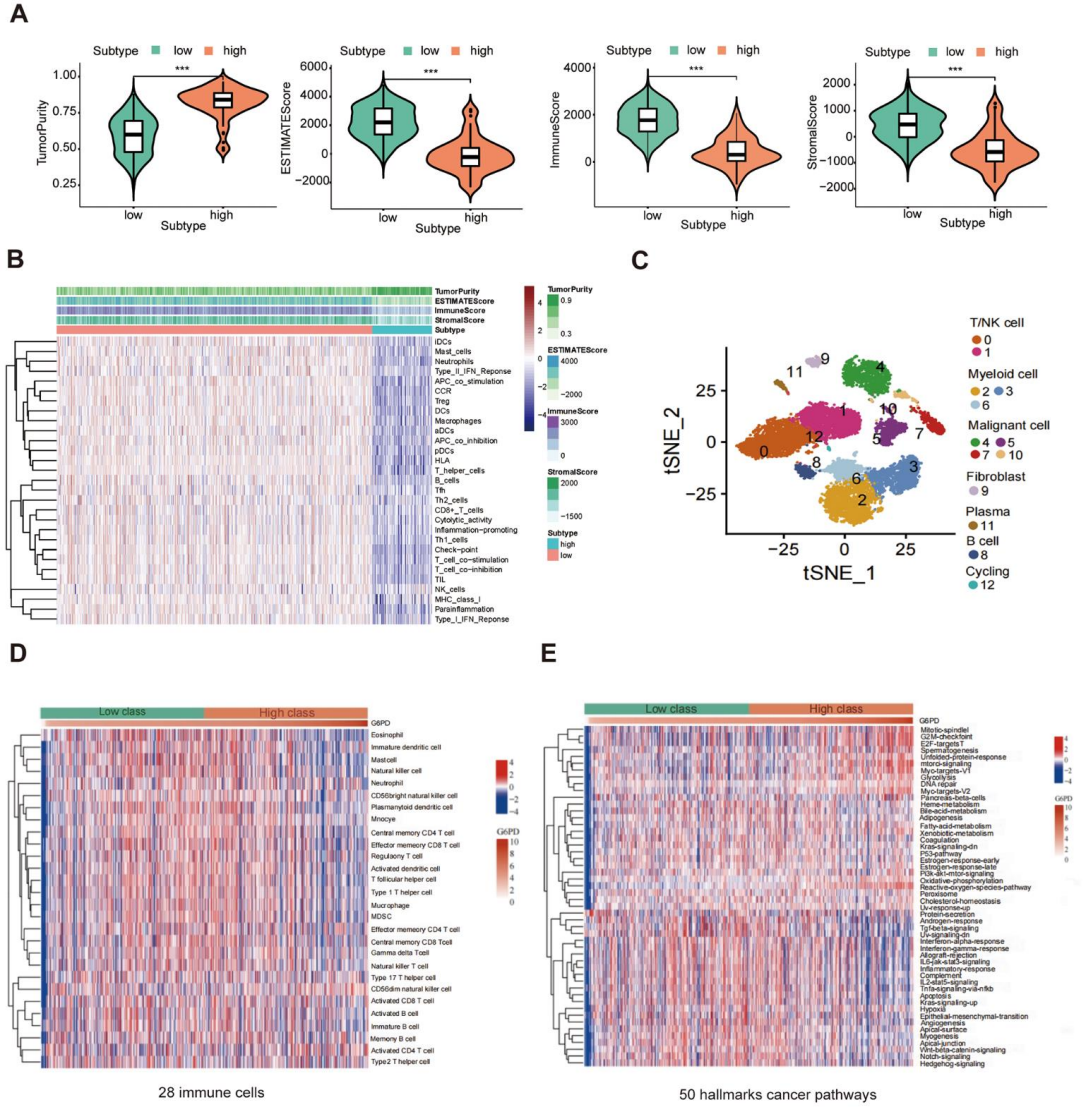
**The correlation between G6PD expression and immune.** (**A**) The ESTIMATE score, immune score, and stromal score between G6PD high and low groups were compared. (**B**) Heatmap of the immune cells between G6PD high and low expression group by ssGSEA algorithm. (**C**) Single-cells analysis from the G6PD high and low groups expression. (**D**) The abundance of 28 immune cells in the two groups using ssGESA. (**E**) The hallmarks of cancer pathways related to G6PD.

The results showed that the G6PD low group had greater ESTIMATE score, immune score, and stromal score levels (*P* < 0.001) (Figure 9A). We used the ssGSEA method to compare the TIME landscape in G6PD (Figure 9B). Therefore, we concluded that G6PD was possibly connected with TIME. We used the “Seurat” package to extract scRNA-seq data of LUAD patients from the GSE117570. Then, 13 immune cell clusters were found via the “FindNeighbors” and “FindClusters” functions. We screened the cell markers of the 13 clusters by the “FindAllMarkers” function. Single-cell analysis revealed that G6PD was significantly enriched in malignant cells, implying that malignant cells may cause the poor prognosis of LUAD patients (Figure 9C). ssGESA analysis revealed that most immune cells activity declined in the high-expression group, such as regulatory T cells and mast cells (Figure 9D). Moreover, G6PD was involved in many cancer-related signaling pathways, such as P53 signaling pathways and PI3K AKT signaling pathways (Figure 9E).

### Identification of G6PD mRNA expression in LUAD and hypoxic HPASMCs

**Figure 10:**
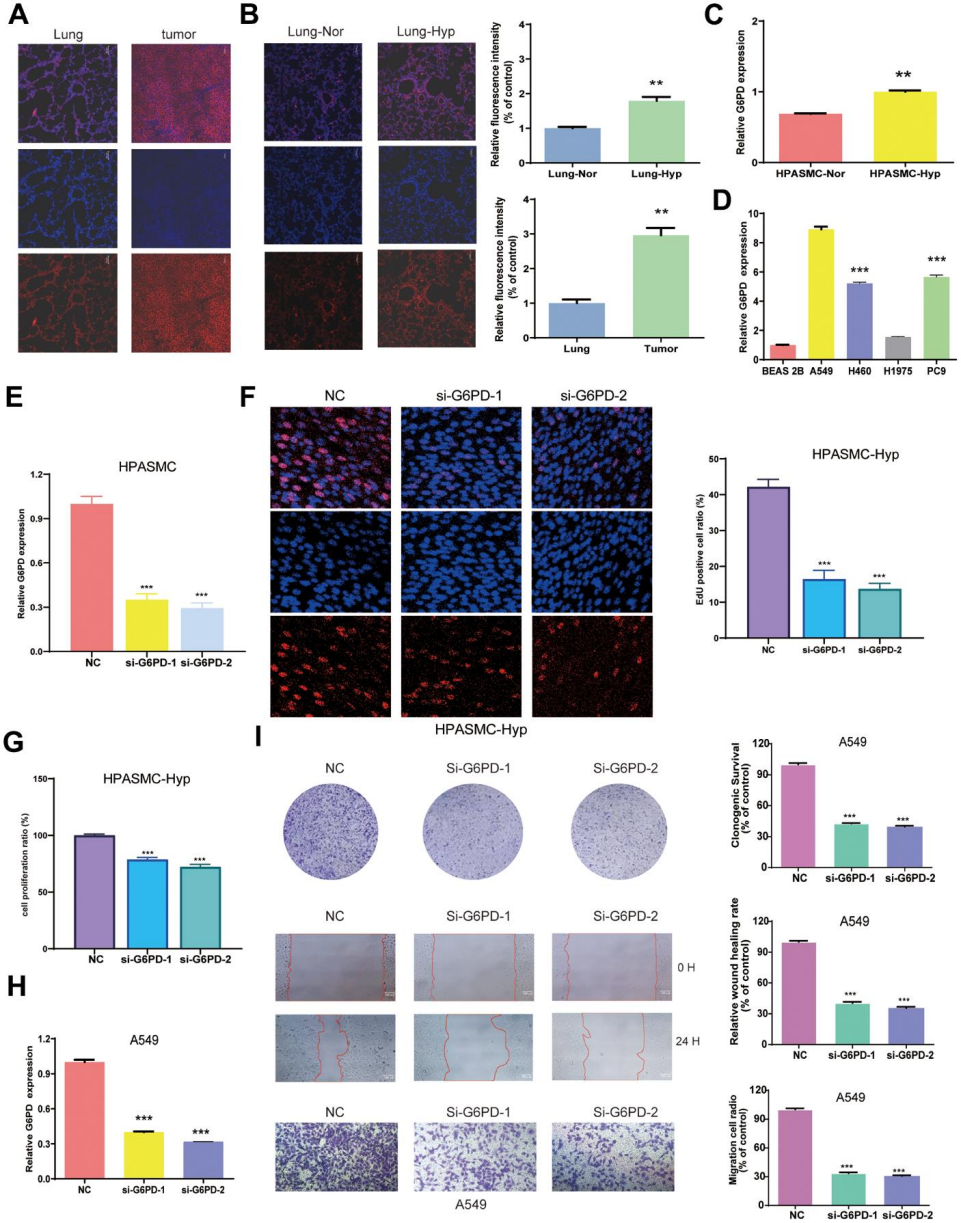
**Identification of G6PD mRNA expression in LUAD and PAH.** (**A**) The expression of G6PD in the tumors and lung organs through immunofluorescence imaging (n = 3). (**B**) The expression of G6PD in hypoxia-induced PAH tissues and normal lung tissue through immunofluorescence imaging (n = 3). (**C**, **D**) G6PD expression levels in normal cells and hypoxia cells (**C**), and LUAD four cell lines (**D**). (**E**) qRT-PCR analysis of G6PD expression in HPASMC transduced with siRNA. (**F**) EDU assay in transduced hypoxia cells. (**G**) CCK8 assay in transduced hypoxia cells. (**H**) qRT-PCR analysis of G6PD expression in A549 cell transduced with siRNA. (**I**) Colony formation assay and transwell assay of migration/invasion ability in transduced A549 cell.

We measured the protein level of G6PD in the tumor tissues and lung organs from BALB/c nude mice by immunofluorescence, and hypoxia-induced PAH tissue and normal lung tissue from the (SD) rats and mice by immunofluorescence. The results indicated that G6PD had a higher level in tumor tissues than lung tissue and a higher level in hypoxia-induced PAH tissues than normal lung tissue (Figure 10A, 10B and Supplementary Figure 2). WB experiment showed that the levels of G6PD increased in tumor tissues than in lung tissue. Similarly, the levels of G6PD increased in hypoxia-induced PAH tissues than in normal lung tissue (Supplementary Figure 3). Subsequently, we performed qRT-PCR, and the result demonstrated that G6PD was significantly over-expressed in LUAD cell lines (A549, H1975, H460, PC9) in comparison to normal BEAS 2B cells (Figure 10D). Meanwhile, G6PD was over-expressed in hypoxia-treated HPASMCs in comparison to normal HPASMCs (Figure 10C).

### G6PD inhibition suppresses A549 cells proliferation, migration, and invasion

We successfully knocked down the G6PD expression in hypoxia-treated HPASMCs by Si-G6PD transfection (Figure 10E). Notably, the EDU and CCK8 experiment revealed that hypoxia-treated HPASMCs proliferation decreased after G6PD knockdown (Figure 10F, 10G). G6PD showed the highest level of mRNA expression in A549 cells among LUAD cells. Therefore, A549 cells were selected for functional analysis. Then, the G6PD expression in A549 cells was successfully knocked down by Si-G6PD transfection (Figure 10H). The results revealed that G6PD knockdown decreased the proliferation ability, clonogenic ability, and migration ability of A549 cells (Figure 10I).

### Candidate drug modules and docking

**Figure 11:**
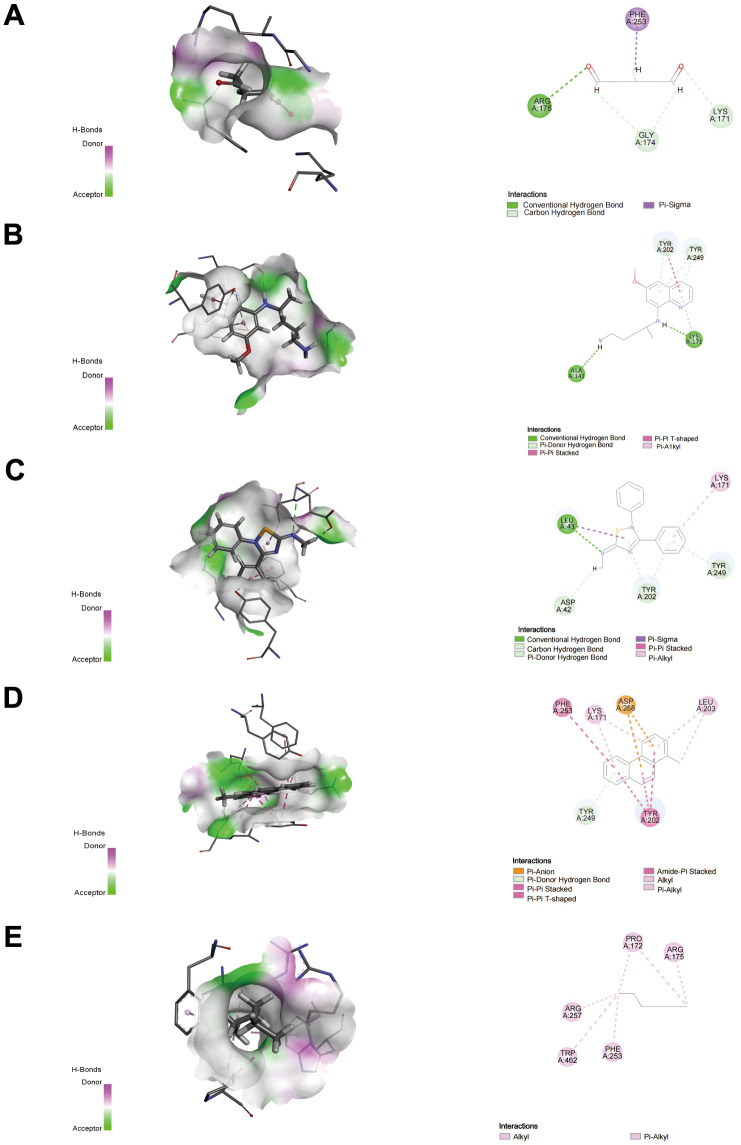
Docking patterns of G6PD interacting with glutaraldehyde (**A**), PRIMAQUINE (**B**), SCH-202676 hydrobromide (**C**), 1-METHYLPHENANTHRENE (**D**), and HEXANE (**E**) as produced using CDOCKER. Nor, normal; Hyp, hypoxia. **P < 0.01, and ***P< 0.001.

We applied the Enrichr platform to identify the potential drug molecules of G6PD based on the DSigDB database. After ranking these drug molecules through the adjusted *P*-value, the top five molecules were screened out. They were glutaraldehyde, PRIMAQUINE, SCH-202676 hydrobromide, 1-METHYLPHENANTHRENE, and HEXANE, respectively, as shown in Table 2. Notably, glutaraldehyde and PRIMAQUINE had a higher combined score. To find the binding affinity of G6PG to the top 5 drug molecules, we performed molecular docking and found that G6PD might have an interaction with these drug molecules, resulting in the -CDOCKER energies of 22.9402, 17.5646, 6.69146, -0.169687, and 17.1697 kcal/mol (Table 3). The results suggested that glutaraldehyde had a stronger binding ability. G6PD had a conventional hydrogen bond with ARG175 of glutaraldehyde, carbon-hydrogen bonds with GLY174 and LYS171, and a Pi-sigma interaction with PHE253. The interaction with PRIMAQUINE includes conventional hydrogen bonds with ALA141 and LYS171 and Pi-donor hydrogen bonds with TYR202 and TYR249. The results of SCH-202676 hydrobromide are related to a conventional hydrogen bond (LEU43), a carbon-hydrogen bond (ASP42), Pi-donor hydrogen bonds (TYR202 and TYR249), and a Pi-alkyl interaction (LYS171). The results of 1-METHYLPHENANTHRENE are mainly associated with Pi-anion (ASP258) and Pi-donor hydrogen bonds (TYR249). The results of HEXANE showed that it interacted with the G6PD proteins via alkyl (Figure 11).

**Table 2:** Top five drug modules associated with G6PD.

**Drugs**	**Adjusted p-value**	**Combined score**	**Related genes**
glutaraldehyde	0.007782212	150029.821	G6PD
PRIMAQUINE	0.007782212	150029.821	G6PD
SCH-202676 hydrobromide	0.007782212	137035.7122	G6PD
1-METHYLPHENA	0.007782212	136099.4703	G6PD
HEXANE	0.007782212	133525.5449	G6PD

**Table 3:** CDOCKER analysis of G6PD at active sites.

**Target**	**Compound**	**-CDOCKER energies**
G6PD	glutaraldehyde	22.9402
	PRIMAQUINE	17.5646
	SCH-202676 hydrobromide	6.69146
	1-METHYLPHENA	-0.169687
	HEXANE	17.1697

## DISCUSSION

Lung cancer is one of the leading causes of cancer-related modality, and LUAD is the most prevalent subtype of primary lung cancer with an increase in incidence [41]. PH is a common comorbidity in lung cancer patients and has a significant impact on clinical outcomes. The prevalence of PH may be higher in lung cancer patients, which contributes to lower survival rates [42]. Dasatinib, a tyrosine kinase inhibitor treating the tumors, may cause PAH in susceptible populations by induction of endothelial damage and dysfunction [43]. The pathogenesis of PAH is related to Mesenchymal and Inflammatory Cell Metabolic [44]. Moreover, cell proliferation and apoptosis resistance in LUAD and PAH may be linked to the inhibition of oxidative metabolism, mitochondrial dysfunction, and H2O2 production [45]. However, few studies are exploring the links between LUAD and PAH at the genetic level. Using the WGCNA, we investigated the common mechanistic underpinnings of LUAD and PAH in this work.

In the current work, we used WGCNA to identify 263 genes in common between LUAD and PAH, 52 of which overlapped with GeneCards disease genes. GO enrichment analysis revealed that the monosaccharide biosynthetic process, membrane raft, and endoribonuclease activity, producing 5’- phosphomonoester were significantly enriched. And these genes were involved in carbon metabolism, arginine biosynthesis, and biosynthesis of amino acids by KEGG analysis. ERLIN2 is a lipid raft-related protein in the endoplasmic reticulum that may be a prognostic biomarker for LUAD relevant to immune infiltration [46]. The fructose 1,6-bisphosphate is a crucial step in cancer metabolic reprogramming which is related to LUAD patients’ prognosis [47]. As part of metabolic reprogramming, carbon metabolism is not only related to cancer progression but also a dysregulated pathway in PAH [48, 49]. Then, we constructed the PPI network analysis, the results revealed that 7 hub genes (G6PD, NIPBL, SHMT2, RNASEH2A, RECQL4, FEN1, and GPT) were linked to LUAD and PAH.

We verified seven hub genes in the discovery and validation cohort to further identify the key gene for LUAD and PAH. Then, we performed RT-PCR. Interestingly, our findings revealed the G6PD gene is upregulated in both LUAD and PAH. The G6PD expression was distinctly upregulated in LUAD cells (A549, H1975, H460, PC9) in comparison with BEAS 2B cells and also upregulated in HPASMCs with hypoxia treatment. Immunofluorescence results showed that G6PD had a higher protein level in tumor tissues than in normal lung tissue and a higher protein level in hypoxia-induced PAH tissues than in normal lung tissue. Notably, G6PD knockdown decreased cell proliferation, migration, and invasion in NSCLC. Our results demonstrated that G6PD could exert an essential role in the prognosis and treatment of the two diseases. The G6PD gene served as the housekeeping gene in all cells [50]. G6PD glycosylation is enhanced in lung cancer, and G6PD activity modulation is a promising therapeutic strategy for lung cancer [51]. G6PD regenerates NADPH and silencing of G6PD and NADPH oxidase 4 (NOX4) resulted in G1/S cell cycle arrest and inhibited melanoma cell activity [52]. Furthermore, G6PD is remarkedly upregulated in several cancers, including lung cancer, hepatocellular cancer, bladder cancer, et al. [53–59]. In addition, ssGSEA analysis of G6PD was related to the immune-related pathway. G6PD has potential oncogenic activity and is related to several cell biological processes in metabolism and redox of tumor progression [60]. G6PD could generate NADPH to promote PAH by the proliferation of PASMCs and cellular trans-differentiation [61, 62]. These studies indicate that G6PD might serve as a shared biomarker between LUAD and PAH.

Several types of research have revealed that immunotherapy is critical to treating cancer patients in clinical [63–65]. More and more pieces of evidence investigated that immunotherapy could effectively extend survival for lung cancer patients [66], and immune checkpoint inhibitors are now the standard therapy for patients with stages III and IV of NSCLC [67]. Immune cell dysregulation and inflammatory factor formation could contribute to PAH, and effective targeted immunotherapy is the key to preventing PAH [68]. G6PD could upregulate the expression of HIF1α to promote CD133+ cell proliferation and contribute to PH [69]. In this study, CIBERSORT analysis demonstrated that individuals in the G6PD high expression group had very significantly lower levels of memory B cells, regulatory T cells, monocytes macrophages M2, resting mast cells, and resting dendritic cells than those in the low group. In comparison to the G6PD low group, there were notably higher naive B cells, plasma cells, follicular helper T cells, macrophages M0, and neutrophils observed. Moreover, ssGESA analysis revealed that most immune cell activities declined in the high-expression group. Single-cell analysis revealed that G6PD was significantly enriched in malignant cells. Significant findings suggest that abnormal pulmonary blood flow could transform the endothelial cells into quasi-malignant cell phenotypes in PAH [70] We also observed that G6PD was negatively associated with the immune, stromal, and ESTIMATE scores in LUAD. The G6PD inhibitor could markedly decrease inflammatory cytokine production of T cells and suppress a respiratory burst of neutrophils [71]. Another study showed that the specific inhibitor of G6PD suppress the M2 phenotype polarization of macrophages [72]. The Redox system of G6PD-NADPH is of great importance for the activation of T cell stability and metabolic regulation of ROS production [73, 74]. G6PD could promote the hypoxia-induced accumulation of macrophages in hypoxic mice lungs [75]. We further identified significant distinctions in immune checkpoints including TIGIT, CTLA4, CD40, CD274, and PDCD1 in the G6PD low and high groups. G6PD regulates granzyme B expression in tumor-specific cytotoxic T lymphocytes as a metabolic checkpoint [76]. Therefore, G6PD is a key gene between LUAD and PAH, which is associated with regulating immunity and TIME.

We further identified relevant molecular drug molecules for the G6PD gene. The top five molecules were glutaraldehyde, PRIMAQUINE, SCH-202676 hydrobromide, 1-METHYLPHENANTHRENE, and HEXANE. Glutaraldehyde and PRIMAQUINE had the higher combined score. Molecular docking revealed that Glutaraldehyde interacted strongly with the protein of G6PD including ARG175 via conventional hydrogen bond, GLY174, and LYS171 via carbon-hydrogen bond, and PHE253 via Pi-sigma. A previous study reported that the nanoparticles which could inhibit tumor cells proliferation *in vitro* were prepared by Glutaraldehyde crosslinking with glutaraldehyde crosslinking with paclitaxel (PTX) loaded and combined with PEG 400 [77]. Glutaraldehyde-modified allergen extracts could disrupt IgE-reactive epitopes and reduce the stimulatory capacity of T cells [78]. These results demonstrate that Glutaraldehyde is a potential immune-related drug molecular for targeting both LUAD and PAH.

However, our study is still subject to some limitations. First, the differences in gene information in the open database might cause WGCNA results bias. Second, experimental studies were required to further investigate the common mechanism of LUAD and PAH. In addition, the PAH model constructed after 24-hour hypoxia treatment and the lung cancer model constructed using human A549 cells in this study cannot represent the chronic process of PAH complicating lung cancer. In the future, our research will first select female nude mice to establish xenograft tumor model. After tumor formation, nude mice were exposed to atmospheric hypoxia (10% oxygen environment) for 3 weeks to establish a more accurate model of PAH complicated with lung cancer for further study. Meanwhile, according to the literature, PASMC isolated from normal donor lung tissue was exposed to conditioned media derived from tumor cell co-culture to establish an *in vitro* model of PAH complicated with lung cancer [71]. Third, we do not have experiments to determine which stage of lung cancer G6PD is more effective. It reported that the levels of G6PD were increased significantly in all stages (I, II, III and IV) of lung cancer when compared with the normal tissues. Nevertheless, there was no significant difference in the levels of G6PD among different stages [51]. Of course, we will continue to conduct research in the following papers. We will collect the blood and tissues of lung cancer patients at different stages for G6PD detection and analysis, and detect which stage of the development of LUAD is more important for G6PD expression. At last, there is no direct evidence that G6PD influences prognosis by immune infiltration, and the mechanisms remain unknown. We hope, in the future, to employ Flow-based techniques to determine the involvement of G6PD inhibition or overexpression in immune cell distribution *in vivo*. Immunohistochemistry was employed to examine the distribution of immune cells in G6PD knockout nude mice model of PAH complicated with lung cancer.

## CONCLUSIONS

In conclusion, we identified the key gene to reveal the potential pathophysiology of LUAD and PAH and determined that G6PD is an immune-related biomarker and possible therapeutic target for LUAD and PAH patients.

## Supplementary Material

Supplementary Figures

Supplementary Table 1
