# Supplementary material for: G6PD is a prognostic biomarker correlated with immune infiltrates in lung adenocarcinoma and pulmonary arterial hypertension
Source: Aging (Albany NY). 2024 Jan 8;16(1):466–92. doi: 10.18632/aging.205381 (PMC10817399; doi:10.18632/aging.205381)
Supplement: Supplementary Figures [file aging-16-205381-s001.pdf]

SUPPLEMENTARY FIGURES

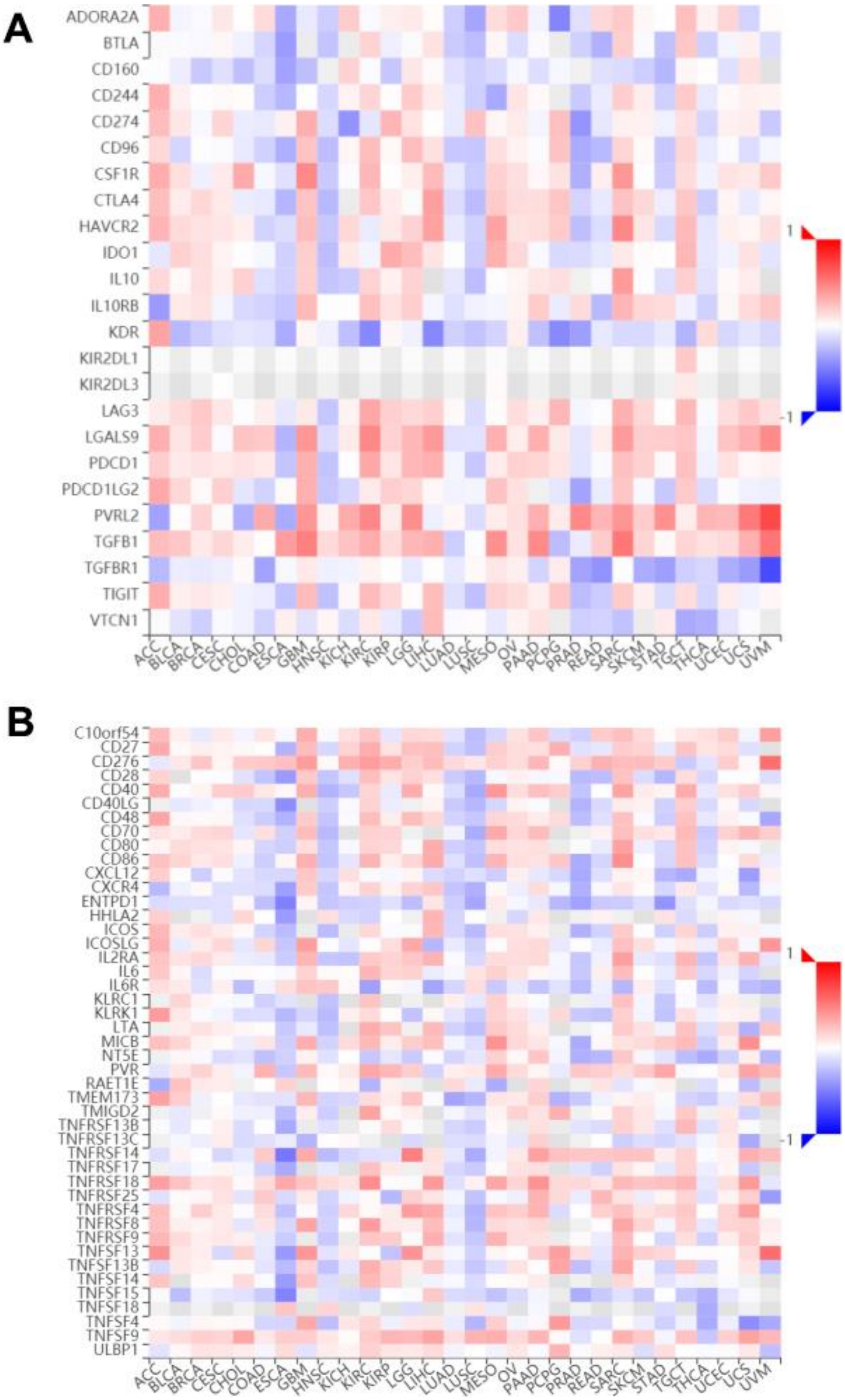

Supplementary Figure 1. The relationship between G6PD expression and pan cancer immunomodulators. (**A**) The relationship between G6PD expression and pan-cancer immunoinhibitor. (**B**) The relationship between G6PD expression and pan-cancer immunostimulator.

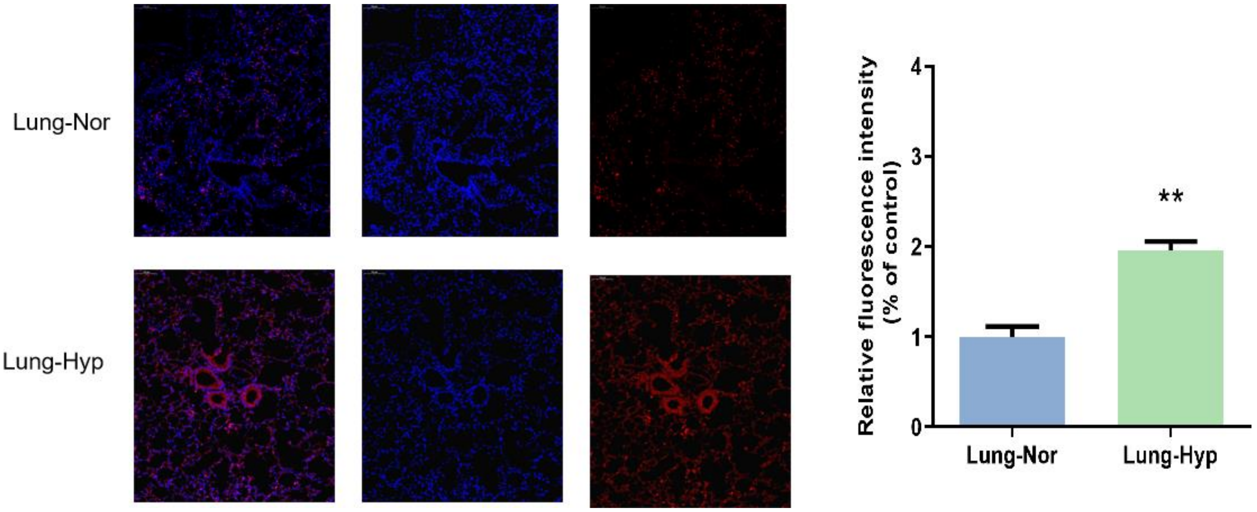

Supplementary Figure 2. The expression of G6PD in hypoxia-induced PAH tissues and normal lung tissue of mice through immunofluorescence imaging (n = 3).

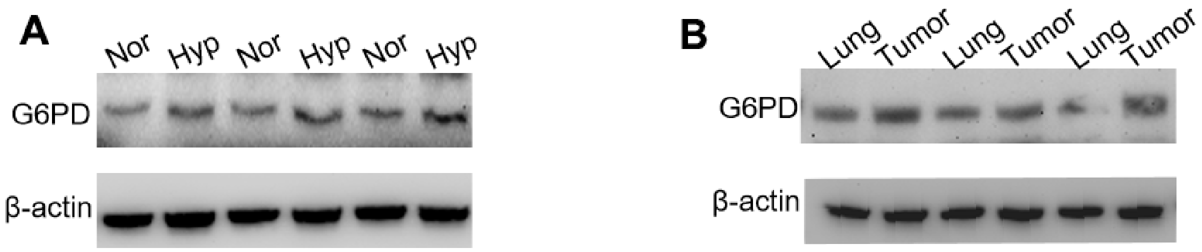

Supplementary Figure 3. The expression of G6PD in hypoxia-induced PAH tissues and normal lung tissue through WB analysis. (**A**) the levels of G6PD increased in tumor tissues than in lung tissue. (**B**) the levels of G6PD increased in hypoxia-induced PAH tissues than in normal lung tissue.
