# Supplementary material for: G6PD is a prognostic biomarker correlated with immune infiltrates in lung adenocarcinoma and pulmonary arterial hypertension
Source: Aging (Albany NY). 2024 Jan 8;16(1):466–92. doi: 10.18632/aging.205381 (PMC10817399; doi:10.18632/aging.205381)
Supplement: Supplementary Table 1 [file aging-16-205381-s002.pdf]

SUPPLEMENTARY TABLE

Supplementary Table 1. The list of 52 overlapped genes of LUAD and PAH.

ACHE
DGKQ
DYRK1B
GPT
ITGB1
KISS1
UCN
FOXD1
TMPO
CD27
CD79A
CAPG
CDK2AP2
CLCNKB
CRAT
CTSD
EBI3
FTL
G6PD
GBA
NOTCH3
PCK2
PFN1
PGAP1
RAB20
RGS19
RTN4R
RUVBL1
SHMT2
SLC25A1
SUV39H1
TRIB3
EIF4EBP1
FEN1
RECQL4
RNASEH2A
SHOX2
ARG2
BRAF
DICER1
ITGA1
JMJD1C
LIMS1
NDUFAF2

NIPBL
NR2C2
PAWR
PCCB
PKD2
ATP8A1
FANCD2
FANCI
